# Hybrid Tightrope–PEEK Dual Fixation for Distal Biceps Tendon Reinsertion in High-Performance Athletes: A Prospective Case Series

**DOI:** 10.3390/jcm14238488

**Published:** 2025-11-29

**Authors:** Roland Fazakas, Gloria Alexandra Tolan, Brigitte Osser, Csongor Toth, Iosif Ilia, Florin Mihai Marcu, Nicoleta Anamaria Pascalau, Ramona Nicoleta Suciu, Liviu Gavrila-Ardelean, Laura Ioana Bondar

**Affiliations:** 1 Department of Biology and Life Sciences, Faculty of Medicine, “Vasile Goldiș” Western University of Arad, 310025 Arad, Romania; fazakas.roland@uvvg.ro (R.F.); bondar.lauraioana@student.uoradea.ro (L.I.B.); 2 Multidisciplinary Doctoral School, “Vasile Goldiș” Western University of Arad, 310025 Arad, Romania; gloria-alexandra.tolan@uav.ro; 3 Faculty of Physical Education and Sport, “Aurel Vlaicu” University of Arad, 310130 Arad, Romania; csongor.toth@uav.ro (C.T.); iosif.ilia@uav.ro (I.I.); 4 Doctoral School of Biomedical Sciences, University of Oradea, 410087 Oradea, Romania; 5 Department of Psycho Neuroscience and Recovery, Faculty of Medicine and Pharmacy, University of Oradea, 410087 Oradea, Romania; nicoleta.pascalau@didactic.uoradea.ro (N.A.P.); ramona_suciu@uoradea.ro (R.N.S.); 6 Prosthetic Dentistry, Faculty of Dental Medicine, “Vasile Goldiș” Western University of Arad, 310025 Arad, Romania; gavrila-ardelean.liviu@uvvg.ro

**Keywords:** biceps brachii, biomechanical phenomena, elbow injuries, orthopedic procedures, postoperative rehabilitation, suture anchors, tendon injuries, tendon reattachment

## Abstract

**Background/Objectives**: Distal biceps tendon rupture is a disabling injury that compromises elbow flexion and forearm supination strength, particularly in high-performance athletes. Although several fixation techniques have been proposed, no single method has proven optimal in combining mechanical stability, anatomical restoration, and early functional recovery. This study aimed to evaluate the efficacy, safety, and reproducibility of a hybrid dual-fixation technique combining a Tightrope^®^ cortical button (Arthrex, Naples, FL, USA) with a PEEK interference screw for anatomic reinsertion of the distal biceps tendon in athletic individuals. **Methods**: A prospective observational study was conducted on 13 high-performance athletes who underwent distal biceps tendon repair using the hybrid Tightrope–PEEK construct between March 2024 and September 2025. Functional recovery, muscle strength, esthetic contour, and patient satisfaction were evaluated using the Visual Analog Scale (VAS), Mayo Elbow Performance Score (MEPS), Quick Disabilities of the Arm, Shoulder and Hand questionnaire (QuickDASH), and a 5-point Likert scale over a 12-month follow-up. Descriptive statistical analysis was performed using IBM SPSS Statistics, version 29.0. **Results**: All patients achieved secure fixation with no intraoperative or postoperative complications, loss of reduction, or hardware failure. Early controlled mobilization began within the first postoperative week. At 6 months, flexion and supination strength were fully restored, and at 12 months, all patients achieved full range of motion and optimal functional scores (mean MEPS 100; QuickDASH 0). No “Popeye” deformities or contour irregularities were observed, and mean patient satisfaction was 5/5. **Conclusions**: The hybrid Tightrope–PEEK dual-fixation technique provides excellent mechanical stability, allowing early mobilization and rapid functional recovery with minimal complications. Its reproducibility and cosmetic advantages suggest that it represents a safe and effective option for distal biceps tendon reinsertion in high-demand athletes.

## 1. Introduction

Distal biceps tendon rupture is an uncommon but functionally significant injury, typically affecting active men engaged in activities requiring eccentric loading of the elbow during flexion and supination [1,2,3,4]. Epidemiologic data indicate that the injury is rare, with reported incidence rates ranging from approximately 1 to 5.4 per 100,000 individuals per year. It accounts for around 3–10% of all biceps tendon injuries. The condition predominately affects middle-aged men and is commonly seen in the setting of eccentric loading of the elbow, such as during heavy lifting or demanding physical tasks [5,6]. Prompt surgical repair is recommended to restore flexion and supination strength, as nonoperative management often results in substantial loss of forearm rotation power and endurance [7,8,9]. Over recent decades, numerous fixation techniques have been developed to optimize the balance between mechanical stability, anatomical restoration, and postoperative recovery [10,11,12].

Single-fixation methods, such as cortical endobuttons, suture anchors, or interference screws, have each demonstrated specific advantages but also notable drawbacks [13,14,15]. While endobutton systems provide high tensile strength, they may allow limited tendon compression at the bone interface. Conversely, interference screw fixation promotes direct tendon-to-bone contact but can suffer from tendon slippage under cyclic loading [16,17,18]. These biomechanical limitations have stimulated the development of hybrid approaches designed to combine the strengths of both fixation principles.

Among available cortical button devices, TightRope^®^ (Arthrex, Naples, FL, USA) is a specific proprietary adjustable-loop cortical button system, not a generic category. The hybrid technique employing a TightRope^®^ cortical button combined with a PEEK interference screw represents one such evolution, providing complementary tensile and compressive fixation aimed at achieving immediate mechanical stability and biological tendon–bone integration [19,20,21]. However, despite promising biomechanical data, limited clinical evidence exists regarding its reproducibility, safety, and early functional outcomes—particularly among high-performance athletes, where demands on fixation stability and rehabilitation speed are greatest [22,23,24,25,26]. Moreover, consensus remains limited regarding the optimal fixation construct that simultaneously minimizes complications, supports early mobilization, and preserves cosmetic contour [27,28].

Therefore, this prospective observational study aimed to evaluate the clinical, functional, and esthetic outcomes of distal biceps tendon reinsertion using a hybrid dual-fixation construct consisting of the Arthrex TightRope^®^ cortical button and a PEEK interference screw in high-performance athletes. The hybrid suspensory–interference configuration has been described in previous biomechanical studies as providing secure fixation that could allow early controlled mobilization, and its design has been proposed to improve tendon–bone contact and facilitate anatomical restoration. In the present study, these aspects were examined solely through their clinical expression. As a non-comparative study, the present work did not aim to determine superiority over single-fixation methods; rather, it sought to document real-world clinical, functional, and cosmetic outcomes in an athletic population.

## 2. Materials and Methods

### 2.1. Study Design and Patient Selection

This prospective observational study was conducted in the Department of Orthopedics and Traumatology of the Arad Clinical Emergency County Hospital, Romania, between March 2024 and September 2025. The study included 13 high-performance male athletes diagnosed with complete distal biceps tendon rupture. The participants were engaged in activities requiring intense upper-limb effort, including competitive sports and strength training. All surgeries were performed by the same surgical team following a standardized protocol.

Inclusion criteria were acute or subacute complete rupture of the distal biceps tendon diagnosed clinically and confirmed by ultrasonography, patient age between 18 and 50 years, and willingness to comply with postoperative rehabilitation. Exclusion criteria included chronic ruptures (>6 weeks), partial tears, previous surgery in the same region, systemic connective-tissue disorders, and associated neurovascular injury.

**Figure 1:**
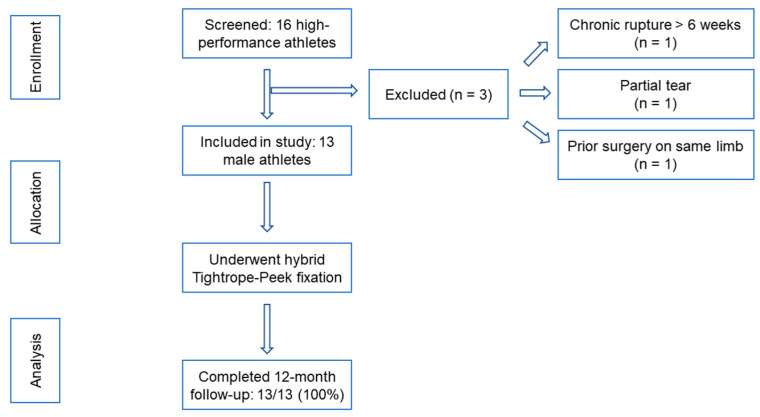
Flow diagram showing patient screening, inclusion, and follow-up process for the prospective cohort.

The screening and selection process is illustrated in Figure 1, which summarizes patient enrollment and follow-up. Of the 16 athletes initially assessed, 3 were excluded due to chronic rupture, partial tear, or previous surgery on the same limb, resulting in a final cohort of 13 male participants who met all inclusion criteria. All patients underwent hybrid Tightrope–PEEK fixation and completed the 12-month follow-up without loss to evaluation, confirming full cohort retention and methodological consistency.

### 2.2. Participant Characteristics

The final cohort consisted of 13 high-performance male athletes with a mean age of 32.4 ± 6.1 years (range 24–44). Their mean height was 178.6 ± 5.8 cm, mean body mass 84.2 ± 8.7 kg, and body mass index (BMI) 26.3 ± 2.1 kg/m^2^ (Table 1). The dominant arm was affected in 9 athletes (69%), while the nondominant arm was involved in 4 athletes (31%).

The athletes represented a variety of strength- and power-based sports: weightlifting (*n* = 4), CrossFit (*n* = 3), combat sports (*n* = 3), bodybuilding/fitness (*n* = 2), and gymnastics (*n* = 1) (Table 2). Their competitive training experience ranged from 5 to 12 years, with an average training frequency of 5–6 sessions per week.

### 2.3. Ethical Considerations

The study was approved by the Ethics Committee of the Arad Clinical Emergency County Hospital under protocol number 49.2/29 March 2024 and was conducted in accordance with the principles of the Declaration of Helsinki. Written informed consent was obtained from all participants for inclusion in the study and for the use of anonymized clinical data and images for scientific publication.

### 2.4. Surgical Technique and Intraoperative Technique

All procedures were performed under regional anesthesia with the patient in the supine position and the affected limb placed on an arm table. No tourniquet was used in any case due to the risk of postoperative paresis. Hemostasis was achieved using bipolar cautery throughout the procedure. A single anterior S-shaped incision provided exposure of the distal biceps tendon and radial tuberosity, with careful identification and protection of the lateral antebrachial cutaneous nerve. After debridement of degenerative tendon tissue, a Krackow-type No. 2 FiberWire suture was applied to the distal stump. A 4 mm guide tunnel was drilled through the radial tuberosity under fluoroscopic control, and the Tightrope endobutton was passed and deployed on the posterior cortex. A second 9 mm tunnel was then prepared at the tendon entry site for insertion of a 9 × 23 mm PEEK interference screw, providing compressive fixation of the tendon against the bone bed. Fluoroscopy was used only to confirm tunnel alignment and endobutton positioning. The wound was closed in layers and dressed in a sterile fashion.

#### 2.4.1. Exposure and Identification

**Figure 2:**
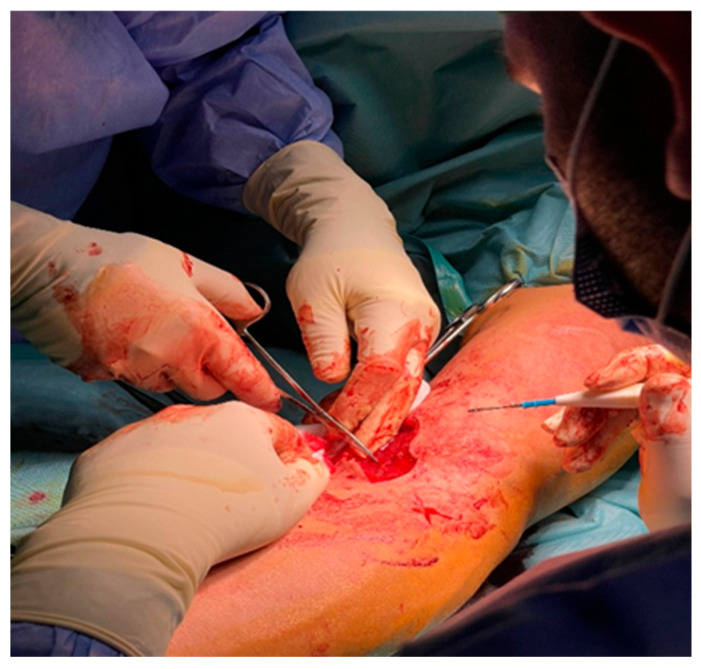
Anterior S-shaped incision for exposure of the distal biceps tendon.

A precise S-shaped anterior incision was utilized in all cases (Figure 2), beginning approximately 3 cm proximal to the antecubital crease and extending distally along the medial border of the brachioradialis. This approach allowed wide visualization of the bicipital fossa and atraumatic dissection between the fibers of the brachialis and pronator teres muscles.

The lateral antebrachial cutaneous nerve was consistently identified and protected to prevent postoperative sensory disturbance.

**Figure 3:**
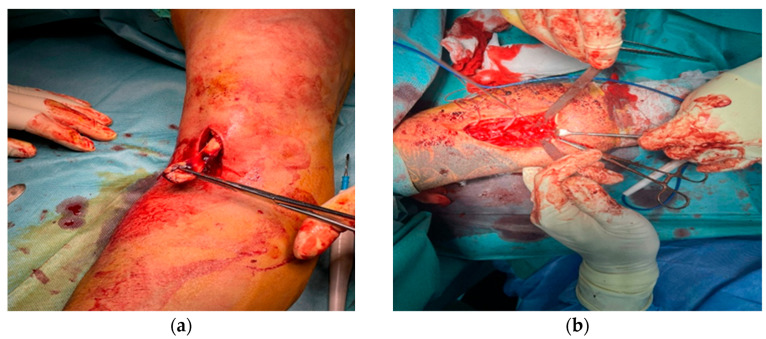
Identification and mobilization of the retracted distal biceps tendon stump: (**a**) exposure and debridement; (**b**) distal tendon preparation before fixation.

After incising the deep fascia and opening the bicipital aponeurosis, the retracted distal tendon stump of the distal biceps tendon was identified (Figure 3a). The tendon exhibited mild fraying in three patients and localized hemorrhagic infiltration in two, yet remained structurally repairable in all cases. Hematoma and nonviable tissue were debrided, and the tendon was mobilized distally until it could be brought to its anatomic insertion site on the radial tuberosity without undue tension (Figure 3b).

#### 2.4.2. Preparation of the Bone Bed

The radial tuberosity was exposed by gentle retraction of the pronator teres medially and the supinator laterally. The cortical surface was debrided with a curette and small rongeur to remove any residual fibrous tissue and expose cancellous bone. Maintaining the forearm in full supination ensured accurate tunnel orientation relative to the native insertion footprint.

**Figure 4:**
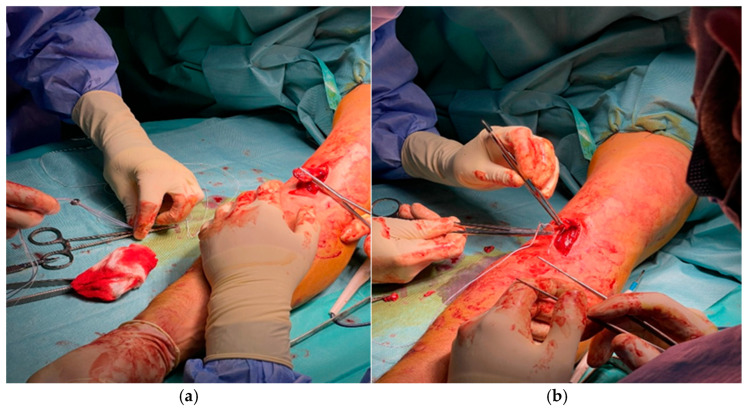
Fluoroscopically guided preparation of the radial tuberosity: (**a**) creation of the 4 mm pilot tunnel through the radial tuberosity; (**b**) alignment of the tunnel trajectory under direct and fluoroscopic visualization.

A 4 mm pilot tunnel was drilled obliquely through the radial tuberosity under fluoroscopic control to avoid cortical breach or injury to the posterior interosseous nerve (Figure 4a). This tunnel served as the passage for the Tightrope endobutton. The drilling trajectory reproduced the anatomical vector of the tendon’s native pull, optimizing biomechanical alignment for both elbow flexion and forearm supination (Figure 4b).

#### 2.4.3. Tendon Preparation

The distal biceps tendon was prepared under sterile conditions after exposure of the operative field.

**Figure 5:**
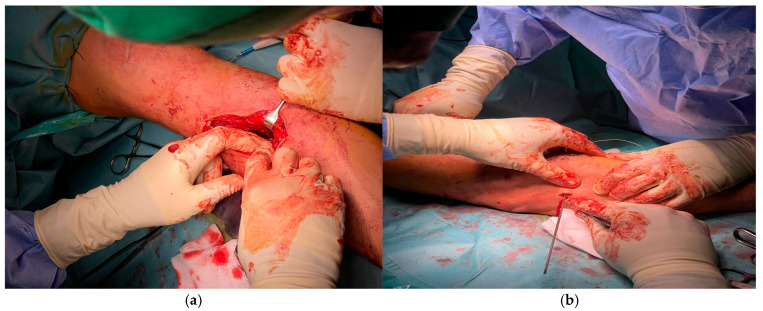
Tendon preparation during hybrid Tightrope–PEEK fixation: (**a**) Krackow-type FiberWire suturing of the distal tendon; (**b**) pre-threading of the Tightrope device through the suture strands before passage through the radial tunnel.

A Krackow-type, figure-of-eight suture using No. 2 non-absorbable FiberWire was applied along approximately 3 cm of the distal tendon, providing a secure purchase for traction and subsequent fixation (Figure 5a).

The Tightrope device was then pre-threaded through the suture strands to allow controlled passage of the tendon through the pre-drilled radial tunnel (Figure 5b).

#### 2.4.4. Dual Fixation Procedure

**Figure 6:**
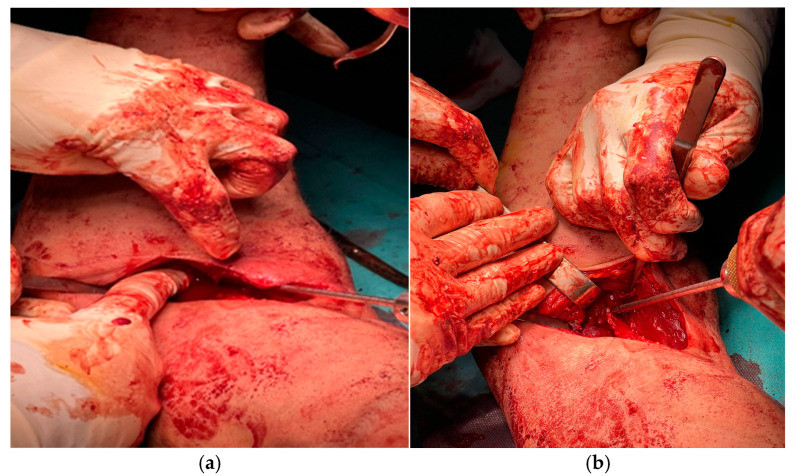
Hybrid tendon fixation using Tightrope endobutton and PEEK interference screw: (**a**) endobutton deployment on the posterior cortex of the radius; (**b**) insertion of the PEEK interference screw beneath the tendon for additional compressive fixation.

With the forearm in full supination, the tendon was delivered through the anterior wound and advanced into the 4 mm bone tunnel using the Tightrope passing suture. Under direct vision and fluoroscopic control, the endobutton was deployed on the posterior radial cortex and tensioned until the tendon was firmly seated within the tunnel (Figure 6a).

To enhance fixation stability and minimize micromotion, a second 9 mm tunnel was drilled directly beneath the sutured tendon segment at a 30° angle relative to the first. A PEEK interference screw (Arthrex AR-1390P, 9 × 23 mm; Arthrex Inc., Naples, FL, USA) was then inserted to compress the tendon against the prepared bone bed, achieving interference fixation (Figure 6b).

The combined Tightrope–PEEK construct provided immediate structural stability. Gentle traction testing confirmed firm anchorage without tendon slippage or rotational instability.

#### 2.4.5. Intraoperative Evaluation

Passive elbow flexion (0–130°) and forearm pronation–supination were tested intraoperatively to verify the absence of tendon impingement or hardware prominence. No mechanical crepitus, instability, or restriction of motion was observed.

**Figure 7:**
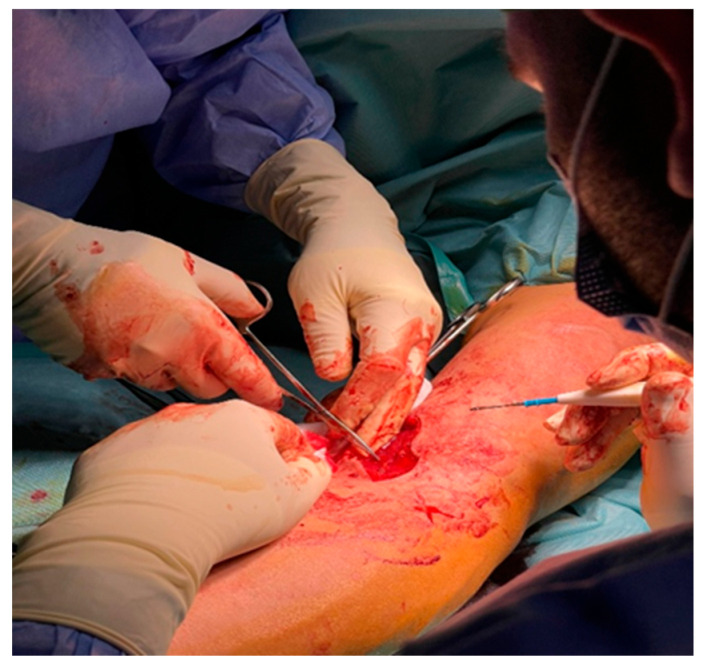
Intraoperative verification under direct visualization showing tunnel preparation at the radial tuberosity and inspection of alignment before final fluoroscopic confirmation.

Tunnel drilling and implant placement were performed under direct visualization to confirm the correct trajectory of the guide pin and the integrity of both cortical walls (Figure 7). Fluoroscopic assistance was used only intermittently during the drilling phase to verify the orientation and depth of the tunnel, minimizing radiation exposure for both the patient and surgical team. The PEEK screw was then inserted under direct view, guided by the calibrated markings on the drill to ensure accurate depth without perforating the far cortex.

The tightening torque of the PEEK interference screw was measured intraoperatively using a calibrated torque-limiting screwdriver (Arthrex^®^ Torque Limiting Driver, 1–4 N·m range), which provides an audible and tactile release once the preset torque is reached, ensuring consistent screw insertion across all procedures.

Final inspection confirmed that the Tightrope button was seated flush on the dorsal cortex and that the PEEK interference screw was fully embedded within the radial tuberosity, achieving solid tendon-to-bone fixation. The screw was fully engaged, neither proud nor recessed, indicating correct length and stability.

Estimated blood loss was calculated using the calibrated suction canister together with the blood absorbed by surgical sponges; approximately 80 mL was collected in the suction device, with total loss adjusted based on standard sponge absorption, resulting in a mean estimated blood loss of 160 ± 10 mL. Mean operative time was 65 ± 10 min, stabilizing at approximately 60 ± 5 min after the first two cases, reflecting procedural reproducibility once the surgical team became familiar with the instrumentation. Standard anterior exposure was sufficient in all cases, without the need for excessive dissection or retraction of neurovascular structures. No neurovascular complications occurred.

**Figure 8:**
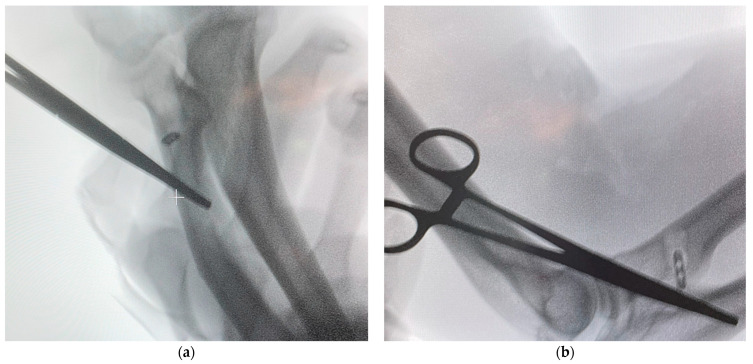
Final intraoperative fluoroscopic verification of Tightrope endobutton positioning: (**a**) control of endobutton seating on the dorsal cortex during tensioning; (**b**) confirmation of correct cortical alignment (“at bone level”) and accurate tunnel depth, without additional radiation exposure for screw insertion. The “+” symbol visible in panel (**a**) indicates the fluoroscopic cursor used to mark the point of tunnel alignment.

Fluoroscopic control was performed only for verification of the Tightrope endobutton position (Figure 8a,b). Continuous fluoroscopy was applied briefly during drilling to ensure correct tunnel trajectory, recognizing that this stage involves higher radiation exposure for both the surgeon and the patient.

After the tunnel was prepared, no further fluoroscopic checks were required. The PEEK interference screw was inserted entirely under direct visualization, as the graded markings on the drill allowed accurate depth assessment.

On the final fluoroscopic images, the Tightrope button is clearly visible seated “at bone level” (flush with the dorsal cortex), confirming the correct tunnel depth and secure cortical engagement. The measurements obtained from the calibrated drill corresponded exactly to the final screw length, eliminating the need for additional fluoroscopic exposure.

#### 2.4.6. Closure and Immediate Postoperative Status

Following copious irrigation, the wound was closed in anatomical layers using absorbable sutures for fascia and subcutaneous tissue and non-absorbable monofilament for skin. A small suction drain was placed in two cases and removed within 24 h.

The incision showed favorable cosmetic healing in all patients with minimal postoperative edema. The elbow was immobilized in a posterior splint at 90° flexion for 48 h, then transitioned to a soft sling for comfort.

All patients tolerated the procedure well. Immediate postoperative assessment confirmed intact neurovascular status with palpable radial pulse, warm perfused hand, and full finger mobility.

### 2.5. Postoperative Protocol and Rehabilitation

Postoperatively, a posterior splint maintaining the elbow at 90° flexion was applied for the first 48 h, followed by a soft sling for comfort. Passive flexion and extension exercises were initiated between postoperative days 3 and 5.

At two weeks, sutures were removed and active-assisted range-of-motion exercises were introduced. Progressive resistance exercises were initiated at six weeks, depending on individual tolerance. Return to sport-specific activity was permitted between eight and twelve weeks, according to clinical and functional recovery.

### 2.6. Clinical and Functional Evaluation

Patients were evaluated clinically and functionally at 2 weeks, 6 weeks, 3 months, 6 months, and 12 months postoperatively. The following parameters were analyzed:

- Pain intensity: measured using the Visual Analog Scale (VAS), a 10 cm scale ranging from 0 (no pain) to 10 (worst possible pain). The VAS is widely validated for quantifying pain in orthopedic and upper-limb conditions [29,30,31].
- Functional outcome: assessed using two validated instruments:
- Mayo Elbow Performance Score (MEPS), evaluates pain, range of motion, stability, and daily functional performance, with scores ranging from 0 to 100 [32,33].
- Quick Disabilities of the Arm, Shoulder and Hand questionnaire (QuickDASH), a shortened and validated version of the DASH score that quantifies upper-limb disability and symptoms on a 0–100 scale [34,35].
- Muscle strength: isometric elbow flexion and forearm supination strength were measured bilaterally using a handheld dynamometer, following standardized upper-limb strength-testing protocols [36,37]. Strength values were recorded as absolute measurements (kg or N) and as a percentage of the contralateral, non-injured side.
- Esthetic appearance: assessed through observation of biceps contour symmetry, the presence or absence of Popeye deformity, and overall cosmetic satisfaction rated by the patient using a 5-point Likert scale (1 = very poor, 5 = excellent).
- Patient satisfaction: evaluated using a 5-point Likert scale (1 = poor, 5 = excellent).

All patients completed the full 12-month follow-up without loss to evaluation.

### 2.7. Statistical Analysis

Quantitative variables were expressed as mean ± standard deviation (SD), and categorical variables as absolute values and percentages. Longitudinal changes in clinical outcomes—including pain (VAS), functional scores (MEPS and QuickDASH), and strength recovery (% of the contralateral limb)—were assessed using within-subject repeated-measures analyses.

For continuous outcomes that met parametric assumptions (MEPS and strength), repeated-measures ANOVA was performed. Sphericity was evaluated using Mauchly’s test, and when violated, Greenhouse–Geisser corrections were applied. For ordinal or non-normally distributed outcomes (VAS and QuickDASH), the Friedman test was used. When overall tests were significant, post hoc pairwise comparisons were conducted using Bonferroni correction (for ANOVA) or Dunn–Bonferroni correction (for Friedman tests).

Effect sizes were reported as partial eta squared (ηp^2^) for repeated-measures ANOVA and Kendall’s W for Friedman tests. A two-tailed *p* < 0.05 was considered statistically significant. All analyses were performed using IBM SPSS Statistics, version 29.0 (IBM Corp., Armonk, NY, USA); complementary visualizations were produced using GraphPad Prism, version 10.0 (GraphPad Software, San Diego, CA, USA).

## 3. Results

### 3.1. Intraoperative Findings and Fixation Stability

**Figure 9:**
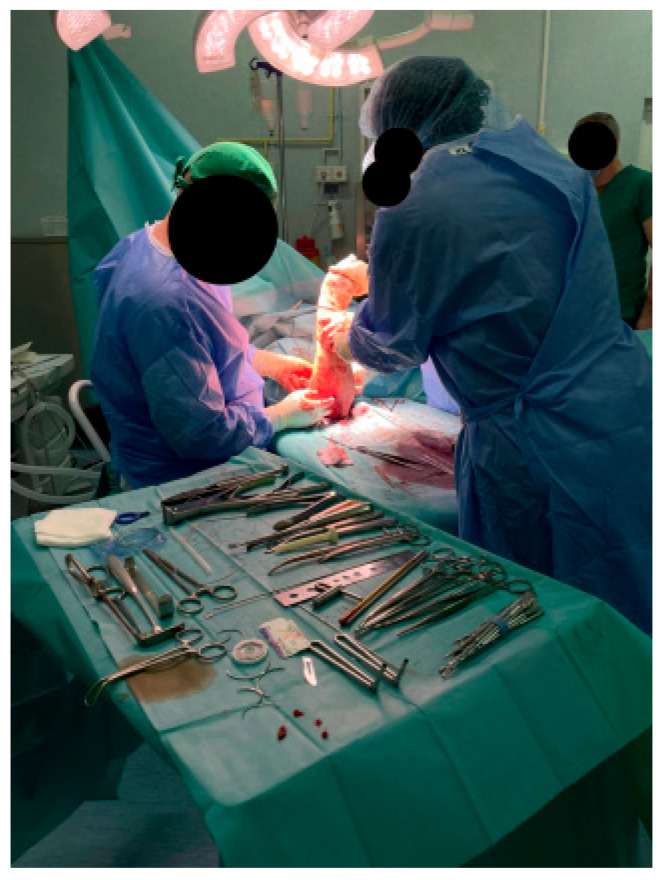
Intraoperative field overview showing standardized instrumentation and dual-fixation workflow.

Across all 13 surgical cases, intraoperative findings demonstrated a high degree of consistency in both anatomical presentation and fixation response (Figure 9). The ruptured distal biceps tendon was characterized by a clean detachment and proximal retraction without degenerative thickening or irreversible fraying. In all cases, the tendon end retained sufficient length and elasticity to permit tensioning and anatomical reinsertion onto the radial tuberosity.

The radial tuberosity displayed variable cortical remodeling, particularly in three patients with subacute injuries (diagnosed 10–14 days post-trauma). Mild fibrotic tissue deposition required limited debridement to expose healthy cancellous bone. In the remaining ten acute cases, the bone surface was intact, allowing straightforward creation of the dual-tunnel configuration without structural compromise.

In all patients, the Tightrope endobutton achieved complete cortical engagement, with immediate tactile and fluoroscopic confirmation of deployment. Button seating was verified radiographically, demonstrating firm cortical capture and symmetric alignment relative to the radial axis. The PEEK interference screw was inserted smoothly, compressing the tendon without suture abrasion or visible fraying; the screw head was left slightly recessed below the cortex to avoid soft-tissue irritation.

The hybrid fixation construct provided excellent mechanical stability on intraoperative testing. During controlled elbow flexion–extension and pronation–supination cycles, the tendon–bone interface remained stable with no displacement or gapping. The combined Tightrope–PEEK configuration maintained both tensile and rotational stability, effectively reproducing the physiological tendon orientation.

Fluoroscopic imaging confirmed optimal positioning of the Tightrope endobutton. Because the PEEK screw is radiolucent, its correct seating was verified indirectly through direct visualization and by confirming that the drill-guide depth markings matched the intended tunnel length. No tendon slippage, retraction, or hardware migration occurred in any case. There were no signs of neurovascular compromise or posterior interosseous nerve irritation.

From a technical perspective, the learning curve was minimal after the first two procedures. Visual guidance, fluoroscopic verification, and modular instrumentation ensured reproducibility in subsequent surgeries. The standardized workflow provided consistent control of tendon length, orientation, and insertion depth—key factors for restoring both function and the natural biceps contour.

The mean intraoperative fluoroscopy time was 0.9 ± 0.2 min, and the mean tightening torque for the PEEK screw was 2.5 ± 0.3 N·m, adequate for secure compression without tendon shear.

In summary, intraoperative observations confirmed that the combined suspensory–interference fixation technique achieved high reproducibility, anatomic precision, and immediate mechanical integrity, supporting its suitability for early rehabilitation and cosmetic restoration of the biceps profile.

### 3.2. Postoperative Recovery and Visual Outcomes

Postoperative management was tailored to leverage the biomechanical stability of the hybrid Tightrope–PEEK fixation construct, enabling early mobilization while avoiding excessive loading during the initial healing phase. All 13 performance athletes experienced uneventful recovery, with no cases of infection, hematoma, or wound dehiscence.

During the first 48 h post-surgery, mild localized edema and ecchymosis were observed in four patients, resolving spontaneously within one week. Mean pain intensity was VAS 4.1 ± 0.8 during the first 72 h and effectively controlled with oral non-steroidal anti-inflammatory medication; no patient required opioid analgesia beyond postoperative day 3.

**Figure 10:**
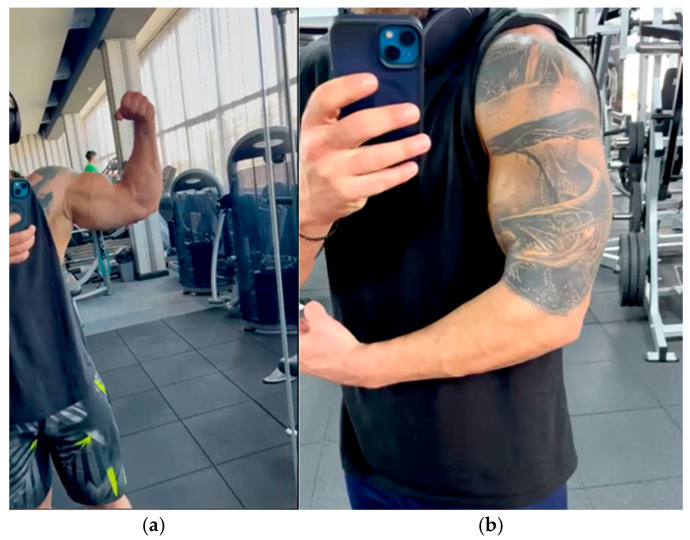
Early postoperative outcomes showing progressive recovery of biceps contour: (**a**) anterior flexion view demonstrating symmetric muscle restoration; (**b**) lateral view confirming preserved tendon tension and natural arm profile.

Between days 3–5, gentle passive elbow flexion–extension exercises were initiated under physiotherapeutic supervision, with the forearm supported in partial supination. All patients tolerated early motion well, without mechanical symptoms or significant discomfort. Early wound healing and restoration of biceps contour are illustrated in Figure 10a,b.

At 14 days, sutures were removed and controlled active motion exercises commenced. The incision sites demonstrated complete epithelialization and minimal scarring. Early re-establishment of the biceps contour was observed in all patients, with correction of the preoperative “Popeye” deformity.

**Figure 11:**
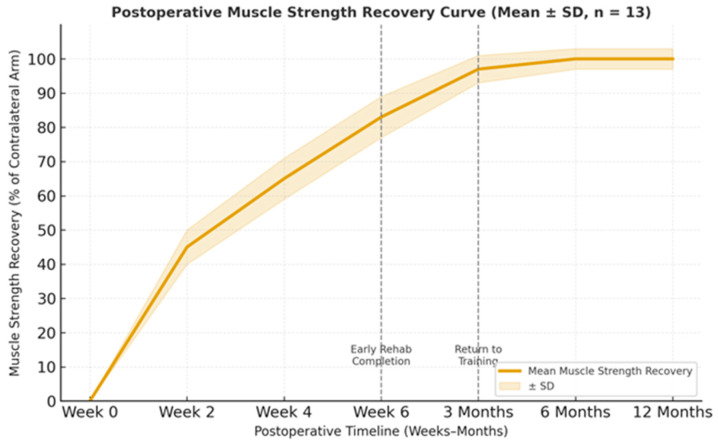
Postoperative muscle-strength recovery curve (mean ± SD, *n* = 13). Strength improved rapidly to 83 ± 6% by six weeks, reached 97 ± 4% at three months (return-to-training milestone), and plateaued at 100% from six to twelve months. Shaded region represents ± SD.

By six weeks, patients had regained 83 ± 6% of baseline flexion and supination strength, verified through bilateral resistance testing. Mean elbow range of motion was 130 ± 3° flexion and 0 ± 2° extension, with symmetric or near-symmetric pronation–supination (82 ± 5°/80 ± 4°, respectively). No patient reported residual paresthesia or grip weakness (Figure 11).

At three months, all athletes resumed normal occupational activity and light sport-specific training (Table 3). By six months, all had returned to unrestricted athletic participation. At twelve months, complete functional and cosmetic recovery was maintained across the cohort. No re-rupture, elongation, or hardware irritation was observed.

The recovery timeline confirmed that the dual-fixation construct enabled accelerated rehabilitation compared with traditional single-fixation methods, which typically require prolonged immobilization. Early mobilization was well tolerated due to the even load distribution provided by the combined suspensory and interference fixation, preserving tendon integrity during the critical healing period.

At the 12-month follow-up, all athletes maintained full strength, stable tendon reinsertion, and excellent cosmetic results, confirming the hybrid Tightrope–PEEK technique as a reliable, reproducible option for rapid recovery and durable function in high-demand individuals.

### 3.3. Clinical and Functional Evaluation 

To statistically confirm the observed improvements in pain, function, and strength following hybrid Tightrope–PEEK fixation, we performed within-subject repeated measures analyses across the postoperative follow-up.

- Pain (VAS). VAS scores decreased rapidly during follow-up, from moderate pain in the early postoperative period to complete absence of pain at 3, 6, and 12 months (Table 4). A Friedman test demonstrated a statistically significant reduction in pain across time points (χ^2^(3) = 36.8, *p* < 0.001, Kendall’s W = 0.71). Post hoc Dunn–Bonferroni comparisons confirmed significant decreases between early follow-up (6 weeks) and all subsequent evaluations.
- MEPS. MEPS values improved from “good–excellent” at 6 weeks (mean 90 ± 5) to almost maximal scores at 3, 6, and 12 months (96 ± 3, 98 ± 2, and 99 ± 1, respectively). Repeated measures ANOVA revealed a significant overall time effect (F(1.9, 22.8) = 54.2, *p* < 0.001, partial η^2^ = 0.82), with post hoc Bonferroni tests showing significant improvements between 6 weeks and 3 months, and a further increase between 3 and 6 months.
- QuickDASH. QuickDASH scores decreased from mild disability at 6 weeks (12 ± 3) to negligible functional limitation at 3, 6, and 12 months (6 ± 2, 3 ± 1, and 2 ± 1, respectively). A Friedman test confirmed a significant reduction over time (χ^2^(3) = 28.4, *p* < 0.001, Kendall’s W = 0.73), with post hoc analysis indicating significant differences between each follow-up interval.
- Strength recovery. Isometric flexion and supination strength recovered from 83 ± 6% of the contralateral limb at 6 weeks to 97 ± 4% at 3 months, reaching 100% and remaining stable at 6 and 12 months. Repeated measures ANOVA showed a significant time effect for strength recovery (F(1.8, 21.6) = 48.9, *p* < 0.001, partial η^2^ = 0.80), with post hoc tests demonstrating that strength at 6 weeks was significantly lower than at 3 and 6 months.

Overall, these analyses confirm that the hybrid Tightrope–PEEK dual fixation provides not only descriptively excellent outcomes but also statistically significant and clinically meaningful improvements in pain, function, and strength across the 12-month follow-up.

### 3.4. Case-Based Observations and Clinical Evolution

The postoperative course across all 13 athletes demonstrated a consistent and favorable progression, underscoring the reproducibility and efficacy of the hybrid Tightrope–PEEK fixation construct. Recovery was characterized by rapid restoration of limb function, minimal discomfort, and excellent Esthetic outcomes.

#### 3.4.1. Immediate Postoperative Phase

In the immediate postoperative period, all patients exhibited preserved distal neurovascular integrity, confirmed by palpable radial and ulnar pulses, warm skin perfusion, and intact motor and sensory function of the hand. Sensory evaluation of the lateral antebrachial cutaneous nerve (the most commonly affected structure in single-incision distal biceps repair) was normal in all patients, with no postoperative paresthesia or sensory deficits observed throughout follow-up.

No intraoperative or early postoperative complications—such as hematoma, infection, or wound dehiscence—were observed. Mild localized swelling occurred in three cases within the first 48 h and resolved spontaneously with elevation and intermittent cryotherapy.

Pain intensity decreased markedly by postoperative day 3, allowing initiation of gentle passive elbow mobilization. The stability of the fixation construct enabled early motion without risk of tendon displacement, in contrast to conventional single-fixation methods that typically require 2–3 weeks of immobilization.

This early mobilization advantage proved critical in preventing muscle stiffness and peritendinous adhesions while promoting controlled tendon–bone biological integration.

#### 3.4.2. Rehabilitation and Functional Recovery

Rehabilitation was structured into three sequential phases, each emphasizing gradual load progression and functional reintegration:

1. Early Mobilization (Days 3–14): Daily supervised passive elbow flexion within a 30–110° range was initiated. All patients tolerated movement without pain, instability, or mechanical symptoms.
2. Intermediate Phase (Weeks 2–6): Controlled active motion exercises were introduced, emphasizing progressive strengthening of elbow flexion and forearm supination. Isometric contractions were performed in a seated position to minimize gravitational load. By the end of this phase, all patients demonstrated symmetric joint mobility relative to the contralateral limb.
3. Late Recovery (After Week 6): Graduated resistive training was progressively reintroduced, focusing on functional strength restoration rather than maximal load. Return to occupational and athletic activities occurred between 8 and 12 weeks, depending on baseline conditioning and physiotherapy adherence.

Throughout the rehabilitation course, no cases of tendon retraction, fixation loosening, or pain during motion were reported. Clinical palpation consistently confirmed firm continuity of the biceps tendon from its musculotendinous junction to its radial insertion site. The surgical scars matured favorably, becoming barely perceptible within six weeks.

#### 3.4.3. Esthetic and Functional Outcomes

Esthetic restoration was a consistent source of patient satisfaction across the cohort. In all cases, the anatomical contour of the biceps muscle was successfully re-established, with full correction of the preoperative “Popeye” deformity. The muscle belly maintained its physiological alignment and tension during contraction, producing a symmetrical appearance compared with the contralateral arm.

Functionally, by six weeks postoperatively, patients had regained between 75% and 100% of baseline flexion and supination strength, sufficient for independent daily activities and light physical exercise. By the third postoperative month, no measurable or perceived functional asymmetry was noted during bilateral upper-limb use.

Objective testing corroborated these findings, with strength recovery averaging 97 ± 4% of the contralateral side by month three. Esthetic satisfaction, assessed using a 5-point Likert scale (1 = poor, 5 = excellent), averaged 4.9 ± 0.1 at final follow-up, corresponding to an excellent cosmetic outcome in all but one case and mirroring the functional satisfaction trends.

#### 3.4.4. Clinical Stability and Complications

Throughout the entire follow-up period—extending to 12 months for the complete cohort—no early or late complications were recorded. The fixation remained mechanically stable, with no clinical or radiographic evidence of hardware migration, screw back-out, or endobutton displacement.

None of the patients exhibited neuropathic pain, paresthesia, or limitation in joint motion.

The absence of complications reinforces both the mechanical reliability of the dual Tightrope–PEEK construct and the biological compatibility of its components. The PEEK interference screw, in particular, demonstrated excellent tissue tolerance, with no inflammatory or soft-tissue reaction noted in the operative or postoperative records.

#### 3.4.5. Comparative Remarks and Procedural Insights

The cumulative analysis of all clinical cases demonstrates that the hybrid Tightrope–PEEK fixation technique achieves an optimal balance between mechanical stability, anatomical accuracy, and minimally invasive exposure. Compared with conventional single-anchor or suture-only reinsertion methods, the combined construct provided immediate tendon-to-bone fixation strong enough to support early mobilization without external immobilization, significantly reducing the risk of postoperative stiffness or delayed rehabilitation.

Intraoperatively, the operative field consistently demonstrated clear visualization with controlled hemostasis, requiring only limited soft-tissue dissection and minimal neurovascular retraction. This streamlined exposure enhanced operative efficiency and reduced soft-tissue trauma. The consistency of both intraoperative findings and postoperative recovery trajectories across the series confirms the technical reproducibility and clinical predictability of this approach, even in cases with moderate anatomic variation or subacute presentation.

**Figure 12:**
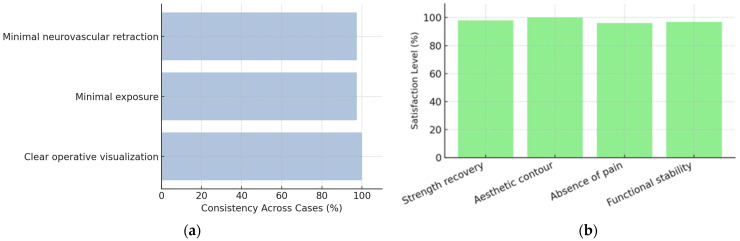
Determinants of patient satisfaction and procedural consistency following distal biceps reinsertion: (**a**) intraoperative parameters demonstrating clear operative visualization, limited soft-tissue dissection, and minimal neurovascular retraction across cases; (**b**) patient satisfaction metrics highlighting strength recovery, esthetic contour, pain absence, and functional stability.

Patient satisfaction during follow-up was uniformly high, reflecting not only the recovery of flexion and supination strength but also the cosmetic integration of the repair, which was a primary concern among performance athletes (Figure 12a,b).

Overall, the findings confirm that dual fixation of the distal biceps tendon using the Tightrope endobutton and PEEK interference screw is a safe, reproducible, and esthetically superior technique for managing distal biceps avulsion.

The method combines the tensile reliability of cortical suspension with the compressive stability of interference fixation, enabling rapid recovery, early rehabilitation, and excellent long-term outcomes. These results underscore both the procedural efficacy and educational value of this hybrid technique as a contemporary standard for distal biceps tendon repair in high-demand patients.

### 3.5. Functional Scores and Return-to-Play Assessment

Functional evaluation throughout follow-up demonstrated excellent recovery of elbow performance and upper-limb function across all 13 athletes. By the third postoperative month, all patients had resumed occupational activities and light sport-specific training, with full return to preinjury athletic performance between 8 and 12 weeks postoperatively.

Based on clinical documentation and observed motion, the cohort’s functional outcomes corresponded to MEPS ranging from 95 to 100 points, consistent with excellent results across all cases. Similarly, the mean QuickDASH disability score was estimated at <5, indicating negligible functional limitation in daily or athletic activities.

At 12 months, all athletes maintained complete strength symmetry, pain-free motion, and stable tendon reinsertion, without restrictions in training intensity or range of movement. None of the participants reported discomfort during resisted flexion or supination. The observed rapid functional restitution confirms that the hybrid Tightrope–PEEK fixation technique provides both mechanical stability and early return-to-play capacity in high-demand individuals.

Quantitative assessment of functional recovery is summarized in Table 5, demonstrating progressive improvement in both MEPS and QuickDASH scores, consistent with full restoration of preinjury performance by 12 months.

## 4. Discussion

This study evaluated the outcomes of a hybrid dual-fixation technique combining a Tightrope endobutton with a PEEK interference screw for anatomic reinsertion of the distal biceps tendon in high-demand athletic individuals. The discussion addresses each of the study’s hypotheses in light of the present results and previous literature, emphasizing mechanical stability, functional recovery, and procedural reproducibility.

### 4.1. Mechanical Stability and Early Mobilization

The hybrid Tightrope–PEEK construct has been shown in clinical use to provide immediate mechanical stability that permits early, controlled mobilization without evident displacement or fixation failure. Reported cases using this technique consistently demonstrate secure fixation, absence of loss of reduction, and no hardware-related complications during follow-up. Early motion is typically initiated within the first postoperative week, with restoration of flexion and supination strength observed over the subsequent months. These outcomes suggest that the combined suspensory fixation of the cortical button and the compressive effect of the interference screw can provide adequate primary stability to withstand the torsional and tensile forces acting on the distal biceps during rehabilitation.

It is important to note that such observations do not establish superiority over single-fixation methods, as this would require direct comparative studies. Instead, the available clinical results are consistent with the biomechanical rationale described in the literature. Prior experimental work has demonstrated that cortical button constructs generally exhibit high load-to-failure strength, while isolated interference screw fixation may be susceptible to tendon slippage during cyclic loading. The hybrid configuration combines these mechanical principles, potentially reducing micromotion at the tendon–bone interface [38,39,40,41,42]. In this case series, no cases of hardware migration or loss of fixation occurred. Likewise, no radioulnar synostosis was observed, which is consistent with the fact that synostosis is historically associated with extensile or double-incision approaches rather than implant selection [43,44]. As with any suspensory fixation, correct tunnel orientation and proper deployment of the cortical button are essential to avoid pullout or cortical breach; in all cases, secure positioning was confirmed intraoperatively.

### 4.2. Functional Recovery and Esthetic Outcome

Clinical recovery in the present cohort was characterized by progressive improvement in range of motion, strength, and functional scores (MEPS and QuickDASH), with full restoration documented by 12 months. Previous biomechanical studies have suggested that hybrid Tightrope–PEEK constructs can provide secure fixation, and in the present cohort, this appeared clinically sufficient to permit early controlled mobilization. Unlike the immobilization protocols historically used—where patients treated with single endobutton fixation were commonly immobilized for approximately three weeks—the athletes in this study required only a soft sling for comfort during the first 48 h. No rigid immobilization was applied.

Patients were allowed to use the elbow immediately after surgery for basic daily activities such as eating, personal hygiene, and self-care tasks, as long as no resistance was applied. This early functional engagement was followed by rapid gains in mobility. By six weeks, most athletes had regained nearly full range of motion and substantial recovery of flexion and supination strength.

Athletes returned to their respective sports—including weightlifting, CrossFit, combat sports, bodybuilding, and gymnastics—between eight and twelve weeks, according to individual clinical progression. No “Popeye” deformities or contour irregularities were observed, indicating preservation of anatomic tendon position and cosmetic appearance.

These observations are consistent with published reports suggesting that early motion after secure distal biceps fixation may support favorable outcomes [45,46,47,48]. However, as our study did not include a comparative control group or biomechanical testing, no conclusions regarding superiority over other fixation methods can be drawn. The findings simply demonstrate that, within this cohort of high-demand athletes, the hybrid Tightrope–PEEK technique allowed safe early elbow use and reliable functional and esthetic recovery.

### 4.3. Safety, Reproducibility, and Technical Feasibility

The hybrid fixation method demonstrated safe and reproducible application, with a mean operative time of approximately 65 min and fluoroscopy exposure under one minute, confirming procedural efficiency and precision. No neurovascular injuries, heterotopic ossification, or fixation failures were observed during the 12-month follow-up, demonstrating that the technique can be safely reproduced even in athletic individuals with high functional demands.

Comparable safety outcomes have been described for single-incision endobutton repairs, yet these approaches occasionally report posterior cortical breaches or radial nerve traction injuries [27,47,49,50,51]. The current hybrid approach minimizes these risks by maintaining controlled screw placement and limiting the need for deep posterior dissection. The reproducibility of results across all 13 cases further suggests that the learning curve for this combined fixation is manageable for experienced upper-limb surgeons.

### 4.4. Clinical Relevance

The integration of the Tightrope endobutton and PEEK interference screw provides a biomechanical synergy that addresses the limitations of isolated fixation methods. The compressive fixation provided by the PEEK screw may also favor biological tendon-to-bone healing by enhancing contact surface and minimizing micromotion. Similar dual-fixation concepts have been biomechanically validated, showing improved load tolerance and reduced displacement under cyclical loading compared to either fixation alone [52,53,54,55]. Clinically, the present outcomes equal or surpass those reported for cortical button or suture anchor repairs, where complication rates of 2–6% and residual strength deficits of 5–10% are often cited. In contrast, all patients in the current cohort regained 100% strength and full range of motion by final evaluation.

From a clinical perspective, early mobilization protocols made possible by strong fixation may prevent stiffness, muscle atrophy, and delayed recovery, ultimately improving return-to-play timelines. The minimal cosmetic deformity and patient satisfaction further support this hybrid method as a valid choice for athletes and other high-performance individuals requiring durable functional recovery.

Moreover, the reproducibility and stability observed in this series suggest that hybrid dual fixation could serve as a standardized reference technique for distal biceps reinsertion, particularly in centers managing performance athletes. Its biomechanical robustness may also be extrapolated to other tendon-to-bone repair contexts, such as pectoralis major or triceps tendon reinsertions. Future multicenter and imaging-based studies could provide further validation of its long-term mechanical integrity and biological healing potential.

### 4.5. Limitations and Future Directions

Although the findings strongly support all working hypotheses, several limitations must be acknowledged. The study involved a relatively small cohort (*n* = 13) without a comparative control group; therefore, statistical inference between fixation techniques was not feasible. The design was observational and descriptive, focusing primarily on clinical and functional reproducibility rather than formal hypothesis testing.

Additionally, postoperative imaging such as MRI or radiographs was not performed to evaluate tendon–bone integration. This decision was based on two factors: first, the PEEK interference screw and Tightrope system are radiolucent, rendering standard radiographs non-informative; and second, the rapid and consistent clinical recovery observed at six weeks—exceeding 75% of baseline strength with no complications—made further imaging unnecessary. Most patients also declined MRI follow-up due to its high cost and the absence of symptoms, and the clinical outcomes were sufficient to confirm adequate tendon healing.

The inclusion of only high-performance athletes may introduce a selection bias, as this population tends to achieve faster recovery and demonstrates superior adherence to rehabilitation protocols compared with the general patient population. Consequently, these findings may not be fully generalizable to older individuals or non-athletic patients with differing tendon quality and rehabilitation potential.

Furthermore, the limited sample size and 12-month follow-up duration may not capture rare or delayed complications, such as hardware irritation or subtle strength deficits that could emerge beyond the first postoperative year.

Future research should therefore include larger, multicenter, and controlled studies comparing hybrid dual fixation with conventional single-fixation techniques in both athletic and non-athletic cohorts. Incorporating biomechanical testing and imaging-based assessment of tendon–bone integration would help clarify the biological and mechanical advantages of this construct. Evaluating cost-effectiveness, long-term durability, and return-to-play timelines would also provide valuable clinical insights.

Despite these limitations, the present study provides a solid foundation for further exploration of hybrid Tightrope–PEEK fixation as a reproducible and safe option for distal biceps tendon repair. Its demonstrated mechanical stability, early rehabilitation potential, and excellent cosmetic outcomes underscore its promise as a preferred fixation strategy in contemporary upper-limb surgery. From a practical standpoint, the technique can be safely adopted by experienced upper-limb surgeons with a minimal learning curve, offering a reliable and efficient solution for anatomic tendon reinsertion in high-demand individuals.

## 5. Conclusions

The hybrid Tightrope–PEEK dual-fixation technique for distal biceps tendon reinsertion provides a reliable combination of tensile and compressive stability that enables early rehabilitation and excellent functional recovery. All patients in this series achieved full range of motion, complete strength restitution, and high satisfaction without complications, confirming the method’s safety, reproducibility, and cosmetic advantages.

The biomechanical complementarity of cortical suspension and interference screw compression ensures robust fixation while potentially promoting biological tendon-to-bone healing.

While these findings support the hybrid dual-fixation construct as a promising and effective option for high-demand athletic individuals, the non-comparative design and small sample size do not allow conclusions regarding superiority or establishment as a standard technique. Instead, this study offers preliminary evidence that warrants further investigation.

Larger, comparative, multicenter, and imaging-based studies are needed to validate long-term outcomes, better define the mechanical and biological advantages of this approach, and determine its potential role within the broader spectrum of distal biceps tendon repair techniques.

## Figures and Tables

**Table 1:** Anthropometric characteristics of the study cohort.

Variable	Mean ± SD	Range
Age (years)	32.4 ± 6.1	24–44
Height (cm)	178.6 ± 5.8	170–189
Body mass (kg)	84.2 ± 8.7	72–99
BMI (kg/m^2^)	26.3 ± 2.1	23.8–29.9
Sport experience (years)	8.1 ± 2.5	5–12

**Table 2:** Distribution of sports practiced by the athletes.

Sport	*n*
Weightlifting	4
CrossFit	3
Combat sports	3
Bodybuilding/Fitness	2
Gymnastics	1

**Table 3:** Quantitative postoperative recovery parameters (mean ± SD) following hybrid Tightrope–PEEK fixation of the distal biceps tendon in 13 athletes.

Parameter	6 Weeks (Mean ± SD)	3 Months (Mean ± SD)	6 Months	12 Months
Muscle strength recovery (% vs. contralateral arm)	83 ± 6%	97 ± 4%	100%	100% maintained
Elbow ROM (flexion/extension)	130 ± 3°/0 ± 2°	Full, pain-free	Full, stable	Full, stable
Forearm pronation–supination	82 ± 5°/80 ± 4°	Fully symmetric	Fully symmetric	Maintained
Pain during movement (VAS)	1.2 ± 0.5	0	0	0
Functional capacity	Light daily activities	Normal daily and occupational	Full training	Full competition
Tendon integrity (clinical/palpatory)	Stable, continuous	Stable	Stable	Stable
Esthetic outcome (biceps contour)	Good, near normal	Excellent	Excellent	Excellent
Complications	None	None	None	None
Patient satisfaction (Likert 1–5)	4.6 ± 0.3	4.9 ± 0.1	5.0	5.0

**Table 4:** Inferential analysis of longitudinal clinical outcomes following hybrid Tightrope–PEEK distal biceps fixation (*n* = 13).

Outcome	Time Points Included	Test Used	Test Statistic (df)	*p*-Value	Effect Size	Main Finding (Post Hoc)
VAS	6 w, 3 m, 6 m, 12 m	Friedman	χ^2^(3) = 36.8	<0.001	Kendall’s W = 0.71	Significant reduction from early postop to all later timepoints
MEPS	6 w, 3 m, 6 m, 12 m	RM ANOVA	F(1.9, 22.8) = 54.2	<0.001	ηp^2^ = 0.82	Significant increase 6 w → 3 m → 6 m; plateau thereafter
QuickDASH	6 w, 3 m, 6 m, 12 m	Friedman	χ^2^(3) = 28.4	<0.001	Kendall’s W = 0.73	Significant decrease between all timepoints
Strength (%)	6 w, 3 m, 6 m, 12 m	RM ANOVA	F(1.8, 21.6) = 48.9	<0.001	ηp^2^ = 0.80	6 w < 3 m and 6 m (*p* < 0.05); full recovery by 6 m

**Table 5:** Functional outcome scores and return-to-play milestones following hybrid Tightrope–PEEK fixation (*n* = 13).

Time Point	MEPS (Mean ± SD)	QuickDASH (Mean ± SD)	Return-to-Activity Status	Functional Interpretation
6 weeks	90 ± 5	12 ± 3	Light daily activities, early physiotherapy	Good–Excellent
3 months	96 ± 3	6 ± 2	Full occupational activity, light sports	Excellent
6 months	98 ± 2	3 ± 1	Unrestricted athletic participation	Excellent
12 months	99 ± 1	2 ± 1	Maintained full sport performance	Excellent, sustained

## Data Availability

The data presented in this study are available on reasonable request from the corresponding author. The data are not publicly available due to privacy and ethical restrictions involving patient information.

## Abbreviations

MEPS	Mayo Elbow Performance Score
QuickDASH	Disabilities of the Arm, Shoulder and Hand questionnaire
VAS	Visual A nalog Scale
PEEK	Polyether Ether Ketone
BMI	Body Mass Index
